# Protein homeostasis in aging and cancer

**DOI:** 10.3389/fcell.2023.1143532

**Published:** 2023-02-16

**Authors:** Xiao-Qiong Chen, Tao Shen, Shao-Jun Fang, Xiao-Min Sun, Guo-Yu Li, Yun-Feng Li

**Affiliations:** Colorectal Surgery, Third Affiliated Hospital of Kunming Medical University, Tumor Hospital of Yunnan Province, Kunming, China

**Keywords:** protein homeostasis, molecular chaperones, ubiquitin-proteasome system, autophagy-lysosomal system, aging, cancer

## Abstract

Aging is a major risk factor for cancer development. As dysfunction in protein homeostasis, or proteostasis, is a universal hallmark of both the aging process and cancer, a comprehensive understanding of the proteostasis system and its roles in aging and cancer will shed new light on how we can improve health and quality of life for older individuals. In this review, we summarize the regulatory mechanisms of proteostasis and discuss the relationship between proteostasis and aging and age-related diseases, including cancer. Furthermore, we highlight the clinical application value of proteostasis maintenance in delaying the aging process and promoting long-term health.

## Introduction

Aging is a complex biological process characterized by gradual and progressive cellular and functional decline. Aging thus remains the greatest risk factor for most chronic disorders, including cardiovascular disease, neurodegenerative disease, and cancer. Protein homeostasis (proteostasis) is essential for preserving normal cellular metabolism and safeguarding physiological function through the proper biosynthesis, folding, trafficking, and degradation of proteins (Morimoto and Cuervo, 2014; Li et al., 2018). Growing evidence indicates that a progressive decline in the capacity to maintain a stable and functional proteome occurs with organismal aging (Vilchez et al., 2014; Kaushik and Cuervo, 2015). Consequently, increased intracellular accumulation of abnormal proteins (e.g., damaged, misfolded, or aggregated proteins) is regarded as an almost universal hallmark of aging, with chronic expression of abnormal proteins resulting in disruption of various biological processes that drive multiple age-related diseases (e.g., Alzheimer’s disease (AD)) (López-Otín et al., 2013). Therefore, ensuring proteostasis is tightly associated with elderly health.

**FIGURE 1:**
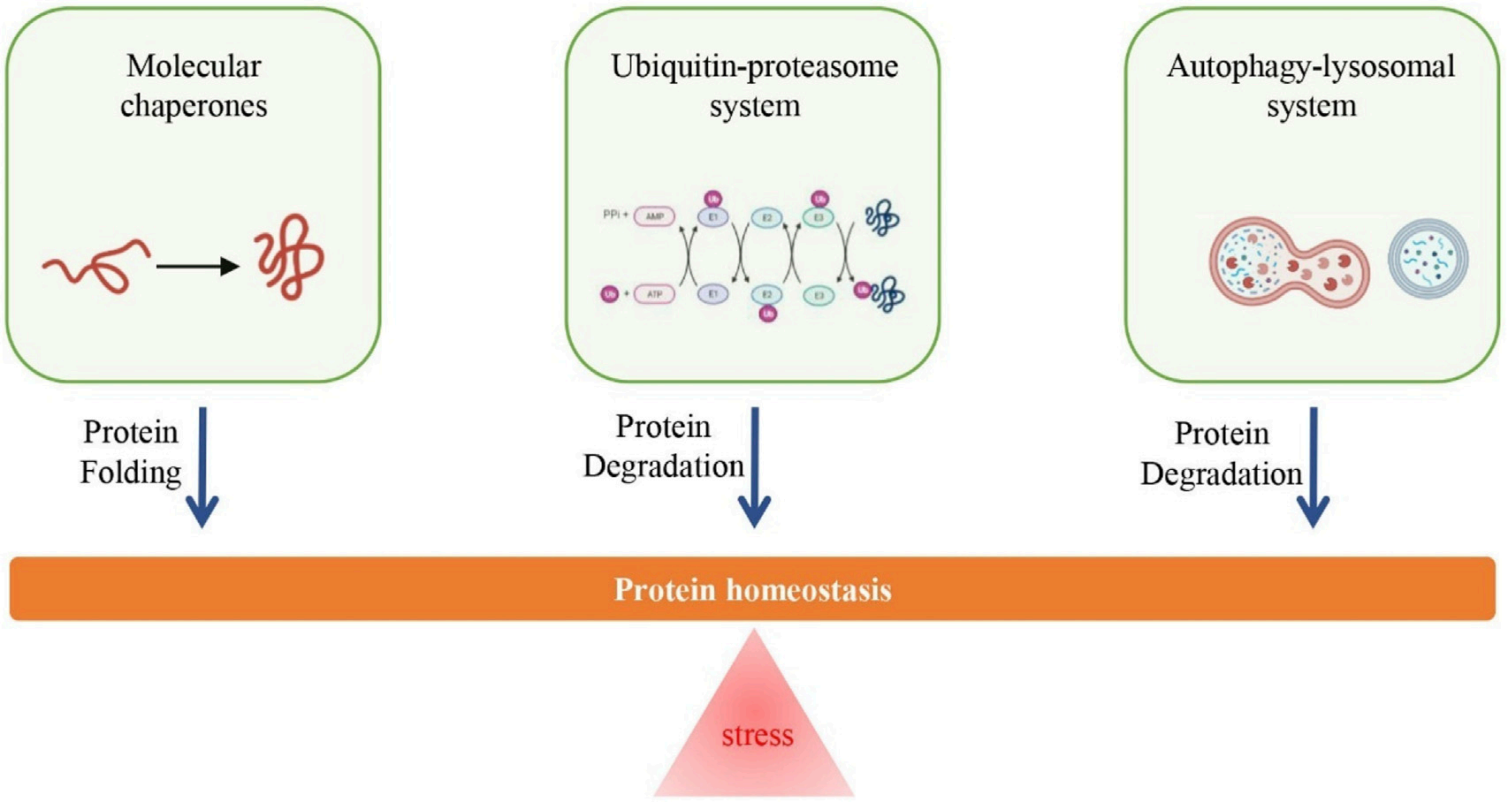
Overview of regulatory mechanisms of protein homeostasis. Long-term chronic stress (e.g., oxidative stress) is an important cause for the loss of protein homeostasis, however, the increase in the function of proteolytic system (i.e., ubiquitin-proteasome system and autophagy-lysosomal system) and repair system (i.e., molecular chaperones) can promote the maintenance of protein homeostasis.

To achieve protein homeostasis, cells have evolved sophisticated quality control mechanisms, primarily consisting of molecular chaperones, ubiquitin-proteasome system, and autophagy-lysosomal system, to promote successful protein folding and eliminate abnormal or misfolded proteins, and thereby adapt to dynamic stress conditions (Kaushik and Cuervo, 2015). Typically, these systems can restore basal homeostasis by rapidly sensing and rectifying the disturbances in proteome; however, long-term chronic stress (e.g., oxidative stress) makes cells difficult to maintain protein homeostasis and proteotoxicity can develop (Figure 1). Various studies have identified functional decline in protein quality control (PQC), including impaired function of the cellular proteolytic mechanisms (i.e., ubiquitin-proteasome and autophagy-lysosome), during aging in different mammals (e.g., human and rat) (reviewed in ref. (Vilchez et al., 2014)). For example, age-related accumulation of intralysosomal lipofuscin (age pigment), likely due to iron-catalyzed oxidative processes, can reduce the degradative function of lysosomes (Brunk and Terman, 2002; Jung et al., 2007). In turn, evidence has also shown that an increase in autophagy-lysosome and/or proteasome activity can extend longevity in diverse organisms, including humans (Chondrogianni et al., 2000; Pérez et al., 2009; Xiao et al., 2018).

Importantly, cancer is considered a disease of aging, but involves an integrated functional network of biological processes related to the regulation of protein homeostasis that dynamically responds to the needs of cancer cells. Cancer cells must adapt to a wide variety of chronic stresses, especially high misfolded protein burdens due to genomic aberrations, and therefore require sustained PQC for survival and proliferation (Chen et al., 2017; Bastola et al., 2018). Thus, modulation of the protein homeostasis network can promote longevity, but at the potential cost of cancer progression. In this review, we summarize the intracellular PQC system and discuss how protein homeostasis functions as a double-edged sword in aging and tumorigenesis. We also highlight the potential of targeting protein homeostasis as a therapeutic strategy for age-related pathologies, including cancer.

## Intracellular regulation mechanisms of protein homeostasis

### Molecular chaperones

Molecular chaperones are structurally diverse and highly conserved ubiquitous proteins that function to maintain protein homeostasis in cells (Arslan et al., 2006). Molecular chaperones, also known as heat shock proteins (HSPs), account for 5%–10% of total proteins in most normal cells (Pockley, 2003). They can specifically and non-covalently bind to the surfaces of interactive proteins and are usually classified according to their functional properties and molecular weight, including major HSP families such as HSP40 (J-proteins), HSP60 (chaperonins), HSP70 (68–78 kDa), HSP90 (85–96 kDa), HSP100 (Clp proteins), and small HSPs (sHSPs, 10–30 kDa) (Hartl et al., 2011). These proteins play important roles in *de novo* protein folding and refolding, protein-complex assembly and disassembly, protein transport across membranes, and protein degradation (Kim et al., 2013; Brandvold and Morimoto, 2015; Shemesh et al., 2021). For example, as an abundant molecular chaperone, HSP90 participates in the folding of a variety of proteins (*viz.* so-called “clients”) involved in protein trafficking, signal transduction, transcriptional regulation, and immunity, utilizing energy generated by adenosine triphosphate (ATP) binding and hydrolysis and interacting with various co-chaperones (Frydman, 2001; Brown et al., 2007; Taipale et al., 2010); moreover, HSP90 can also enhance protein degradation (e.g., oxidized proteins) through the proteasome (Whittier et al., 2004). Likewise, HSP70 also participates in the maintenance of protein homeostasis by interacting with many proteins to facilitate the prevention of protein misfolding or the degradation of damaged proteins (Seo et al., 2016; Garbuz et al., 2019). Taken together, HSPs play important roles in responding to various stresses (e.g., high temperature) and facilitating cellular survival during life.

### Ubiquitin-proteasome system

The ubiquitin-proteasome system is a key player in intracellular protein degradation and turnover, and thus plays an essential role in cellular protein homeostasis (Glickman and Ciechanover, 2002; Raaben et al., 2010; Gong et al., 2016). Approximately 80% of cellular proteins can be degraded by the ubiquitin-proteasome system (Meyer-Schwesinger, 2019). These proteins are associated with many biological processes, including cell cycle progression, apoptosis, gene transcription and translation, cell survival, and antigen presentation (Raaben et al., 2010; Park et al., 2020). This degradation system requires the conjugation of ubiquitin (small, highly conserved protein of 76 amino acids) to target proteins by the sequential action of three enzymes: ubiquitin-activating enzyme (E1), ubiquitin-conjugating enzyme (E2), and ubiquitin ligase enzyme (E3) (Shu and Yang, 2017). Briefly, this degradation process is initiated by the formation of E1-ubiquitin thioester bonds between the active Cys residue site of E1 and C-terminal Gly carboxyl group of ubiquitin through ATP-dependent reactions. Thioester-linked ubiquitin is then transferred to the catalytic Cys in E2, resulting in the formation of an E2-ubiquitin thioester-linked conjugate. After this, E3 promotes the transfer of ubiquitin from the E2-ubiquitin conjugate to the Lys residues within the different substrate proteins by recognizing their specific motifs. Finally, the targeted proteins with polyubiquitin chains are recognized and degraded by the proteasome (Haas et al., 1982; Gong et al., 2016; Shu and Yang, 2017). During this progress, ubiquitin is the “signal” for protease cleavage of the protein, with various chains of ubiquitin molecules labeling different abnormal proteins. Furthermore, ubiquitylation is a reversible process, catalyzed by a series of deubiquitylating enzymes, in which ubiquitin molecules removed from protein substrates can be released and recycled (Finley, 2009; Qiu et al., 2022).

### Autophagy-lysosomal system

The autophagy-lysosomal system is another important mechanism in cellular homeostasis for the degradation and recycling of cytoplasmic components, such as defective proteins and organelles (Mariño et al., 2011; Li et al., 2018). This system participates in the regulation of multiple biological processes, including cell growth, differentiation, remodeling, and senescence (Schuck, 2020). Depending on how excess or damaged cytoplasmic material is delivered to the lysosomes, autophagy can be classified as macroautophagy, microautophagy, or chaperone-mediated autophagy (CMA) (Mariño et al., 2011). Macroautophagy is a dominant form of autophagy (Miller and Thorburn, 2021; Griffey and Yamamoto, 2022), whereby superfluous and damaged proteins/organelles as sequestered in nascent double-membrane autophagosomes that fuse with lysosomes for degradation (Feng et al., 2014). The degradation products are released from the lysosomes into the cytosol, with the macromolecular constituents recycled into metabolic and biosynthetic pathways to maintain cell viability under unfavorable conditions and to protect the cell during stress (Feng et al., 2014; White et al., 2015). Unlike macroautophagy, microautophagy involves the direct engulfment of material by lysosomes through invaginations or protrusions of the lysosomal membrane (Schuck, 2020). Microautophagy is responsible for the maintenance of organelle size, membrane composition, cell survival under nitrogen restriction, and transition from starvation-induced growth arrest to the logarithmic growth phase (Roberts et al., 2003). CMA is a form of selective autophagy responsible for the degradation of 30% of cytosolic proteins under prolonged nutrient deprivation (Dice, 2007; Bourdenx et al., 2021). CMA is distinct from other types of autophagy in that the substrate protein is directly translocated across the lysosomal membrane for degradation (Schneider et al., 2015). In CMA, substrate proteins are selectively targeted to lysosomes and translocated into the lysosomal lumen through the coordinated action of chaperones located on both sides of the membrane and the dedicated protein translocation complex lysosomal-associated membrane protein 2A (LAMP2A) (Cuervo and Wong, 2014; Bourdenx et al., 2021).

## Protein homeostasis in aging and longevity

Impaired protein homeostasis, characterized by the accumulation of protein aggregates, is a crucial hallmark of aging and age-related diseases, including neurodegeneration (López-Otín et al., 2013; Hipp et al., 2019). Various internal and external stresses that persist throughout life can disrupt protein homeostasis in organisms. Here, we mainly discuss oxidative stress, generated by redox imbalance between the production of reactive oxygen and nitrogen species (ROS and RNS, respectively) and antioxidant defenses, given its importance as a driver of oxidized protein accumulation in senescent cells and aged organisms (Squier, 2001). Multiple studies have observed increases in oxidized proteins in different tissues (e.g., brain and heart) of aged animals, including humans (Granold et al., 2015). Oxidative modification of proteins leads to changes in protein structure, including oligomerization, protein misfolding, and protein backbone fragmentation (Zavadskiy et al., 2022). In turn, oxidative damage to proteins plays a crucial role in accelerating aging (Nyström, 2005; Kim et al., 2012). For example, oxidized low-density lipoprotein plays an important role in promoting retinal pigment epithelial cell senescence (Kim et al., 2012).

In general, oxidized proteins can be eliminated by degradation systems and repair mechanisms in younger cells and organisms, with an extensive network involving molecular chaperones, ubiquitin-proteasome system, and autophagy-lysosomal system. However, protein homeostasis network capacity declines significantly with age (Ben-Zvi et al., 2009; Morimoto and Cuervo, 2014). Age-related intralysosomal lipofuscin accumulation and impaired acidification (i.e., pH) are two important causes of reduced lysosomal degradation activity (Brunk and Terman, 2002; Colacurcio and Nixon, 2016). Age-related reductions in certain key regulatory factors, such as LAMP2A, can also disrupt the autophagy-lysosomal process (Cuervo and Dice, 2000). In addition, decreased proteasomal degradation activity can also result from age-related factors, including decreased expression of both non-catalytic and catalytic subunits of the proteasome with aging (Ponnappan et al., 2007). Studies have also linked the accumulation of oxidatively modified proteins to different age-related diseases, such as neurodegenerative disorder and cardiovascular disease (Barnham et al., 2004; Dunlop et al., 2009; Luna et al., 2016). For example, based on redox proteomics, several studies have reported the presence of oxidized/misfolded proteins in different brain regions of patients with AD (Sultana et al., 2006), with oxidization of glycolytic and TCA enzymes leading to a decrease in ATP production and progression of AD (Tramutola et al., 2017). There are also studies supporting that the aberrant expression of genes involving to the maintenance of protein homeostasis plays an important role in the deposition of Aβ peptide and tau protein in the brains of AD patients (Freer et al., 2016; Rishika et al., 2017). In addition, oxidized albumin can induce endothelial injury and increase the risk of cardiovascular disease in elderly individuals (Luna et al., 2016). Taken together, these findings highlight the crucial role of cellular homeostasis maintenance, especially the elimination of oxidized proteins, in healthy aging organisms.

Accordingly, increasing evidence suggests that enhancement of protein homeostasis network capacity can extend lifespan or promote longevity in various species, such as yeast, worms, flies, mice, and humans (Pyo et al., 2013; Schumpert et al., 2014; Chondrogianni et al., 2015; Madeo et al., 2015; Xiao et al., 2018). For example, overexpression of molecular chaperones (e.g., HSP70, HSP16) can lead to an increase in lifespan (Walker and Lithgow, 2003; Schumpert et al., 2014). Likewise, upregulation of certain autophagy-lysosomal pathway genes is linked to lifespan extension (or longevity promotion). Activation of transcription factor EB (TFEB), a key regulator driving autophagy and lysosomal gene expression, is associated with healthy longevity (Lapierre et al., 2013). Overexpression of the *Atg5* gene, which is essential for autophagosome formation, can extend the median lifespan of mice (Pyo et al., 2013). In addition, a summary of key genes required for protein homeostasis maintenance and longevity promotion is provided in Table 1. Furthermore, centenarian-based evidence suggests that increased autophagy-lysosomal activity is an important mechanism of healthy aging and longevity in humans (Xiao et al., 2018). Studies have also shown that overexpression of proteasome subunits can increase lifespan (Chondrogianni et al., 2015). Interestingly, a growing body of research suggests that protein homeostasis is a key mechanism linking certain interventions to longevity promotion and health improvement, with most demonstrating autophagy-activating properties (Kaushik and Cuervo, 2015). For example, calorie restriction, physical exercise, mTORC1 inhibition, sirtuin 1 (SIRT1) activation, spermidine treatment, and p53 suppression, interventions known to extend lifespan and/or healthspan, can enhance protein homeostasis network capacity, although probably through different mechanisms (Mizushima and Komatsu, 2011; Madeo et al., 2015; Ulbricht et al., 2015; Plaza-Zabala et al., 2017). Thus, exploring how to mitigate age-related decline in PQC capacity should provide new perspectives for achieving healthy aging and longevity.

**TABLE 1:** Examples of proteostasis-related genes linked to organismal longevity. (All gene names were in lower case and only one representative study for each gene was listed here).

Gene	Function	Association with longevity	Species	Refs
***atg-18***	Phagophore formation	Mutational inactivation of *atg-18* reduce lifespan	*C. elegans*	Tóth et al. (2008)
***wwp-1***	E3 ubiquitin ligase	*wwp-1* is required for the extension of lifespan by dietary restriction	*C. elegans*	Carrano et al. (2009)
***hsf-1***	A master regulator of HSP expression	Overexpression of *hsf-1* ubiquitously in somatic cells extends lifespan	*C. elegans*	Morley and Morimoto (2004)
***atg-8a***	Autophagosome formation	Neuronal overexpression of *Atg-18a* extends adult lifespan	*D. melanogaster*	Simonsen et al. (2008)
***hsp27***	Heat shock protein	Neuronal overexpression of *Hsp27* extends lifespan	*D. melanogaster*	Liao et al. (2008)
***parkin***	E3 ubiquitin ligase	Ubiquitous or neuron-specific upregulation of *Parkin* extends lifespan	*D. melanogaster*	Anil et al. (2013)
***becn1***	Autophagosome formation	Mutation in *Becn1* decreases its interaction with BCL2 leads to higher levels of basal autophagic flux and extends lifespan	*M. musculus*	Fernández et al. (2018)
***atg-5***	Autophagosome formation	Ubiquitous overexpression of *Atg5* enhances autophagy and extends lifespan	*M. musculus*	Pyo et al. (2013)

## Protein homeostasis and cancer

Although cancer is also an age-related disease, its biological underpinnings are tightly associated with protein homeostasis. Genomic instability and oxidative stress can lead to increased production of damaged and/or dysregulated proteins in cancer cells (Wu et al., 2014; Bastola et al., 2018). To resolve the overwhelming proteotoxic stress, cancer cells require sophisticated PQC mechanisms to maintain a proper protein homeostasis for survival and growth. Accordingly, there is a growing body of evidence supporting the dual roles of PQC mechanisms in the pathogenesis of human cancers through the building and turnover of tumor-promoting/suppressing proteins. Here we mainly summarized the findings on the crucial functions of the three PQC systems (*viz.*, molecular chaperones, ubiquitin-proteasome system, and autophagy-lysosomal system) in cancer progression.

First, HSPs function as the molecular chaperones to mediate proper protein folding, with more likely being oncogenic function. Numerous HSPs (e.g., HSP60, HSP70, HSP90) have been reported to be overexpressed in a wide range of cancers and are indicative of poor patient prognosis (e.g., gastric, liver and breast cancer) (Ciocca and Calderwood, 2005; Zagouri et al., 2012; Li et al., 2014; Wang et al., 2021). HSPs can promote cancer progression *via* different pathways. For example, overexpression of HSP90 can downregulate E-cadherin and promote epithelial-mesenchymal transition (EMT), a key step in tumor metastasis (Hance et al., 2012). HSP90 can also stabilize vascular endothelial growth factor and nitric oxide synthetase in endothelial cells to induce tumor angiogenesis (Sun and Liao, 2004).

Second, as one of the major proteolytic system, autophagy-lysosomal system is tightly associated with cancer development and progression (Rosenfeldt and Ryan, 2009; Yun and Lee, 2018). On the one hand, autophagy is thought to play an important role in promoting cancer cell survival and growth in advanced cancers (Luo et al., 2016; Liu et al., 2018), as it can provide the substrates (e.g., amino acid) for metabolism through the intracellular recycling of damaged or superfluous proteins and then elicit the formation of an adaptive protein homeostasis in cancer cells (White, 2012; 2015). Furthermore, autophagy may contribute to tumor progression by decreasing the levels of some proteins with tumor-suppressing function. For example, the *ATG7* gene, which is overexpressed in invasive bladder cancer tissue, can promote autophagic degradation of the HNRNPD (ARE/poly(U)-binding/degradation factor 1) protein, which, in turn, increases ARHGDIB mRNA stability and bladder cancer cell invasion (Zhu et al., 2019). Study shows that autophagy participates in the degradation of tumor suppressor PP6 (protein phosphatase 6), the level of which correlates with poor prognosis in glioblastoma (Fujiwara et al., 2020). However, on the other hand, autophagy is also considered to be a tumor suppressor mechanism in the early phages of tumorigenesis as it can inhibit tumors by removing oncogenic protein substrates, toxic unfolded proteins, and damaged organelles, thereby maintaining genomic stability (White, 2012; 2015; Xu et al., 2015). Important evidence for the role of autophagy in tumor suppression comes from the depletion of the essential autophagy regulator *BECN1* (Beclin 1, also known as autophagy-related gene 6 (*ATG6*)) in human breast, prostate, and ovarian cancers (Aita et al., 1999; Liang et al., 1999; Choi et al., 2013). Loss of *BECN1* can lead to a reduction in autophagy and increase in cell proliferation (Liang et al., 1999; Qu et al., 2003; Shen et al., 2008). In addition, there is a study showing that autophagy induction can attenuates the Wnt signalling by promoting Dishevelled degradation, which further inhibits the formation of colon cancer (Gao et al., 2010). Autophagy is also required to suppress the accumulation of oncogenic p62 protein aggregates and prevent tumor initiation (White, 2012).

Third, it has been reported that the increased rate of protein turnover in cancer cells also requires the ubiquitin-proteasome system, which subsequently regulates the “quantity” and “quality” of various proteins (Crunkhorn, 2018; Deng et al., 2020; Zhang et al., 2020). That is, in order to adapt the oxidative and proteotoxic stresses during tumorigenesis, cancer cells rely on the ubiquitinating and deubiquitinating enzymes to maintain protein homeostasis and cell viability (Hyer et al., 2018; Harris et al., 2019). In addition, accumulating evidence suggests that proteins encoded by oncogenes and tumor suppressor genes may be targets of ubiquitination, that is, ubiquitin-mediated proteasomal degradation could either activate or deactivate the tumorigenic pathways. For example, there are studies showing that the protein level of tumor suppressor p53 can be reduced by its ubiquitination and proteasome degradation, resulting in poor survival and prognosis in cancer patients (e.g., colorectal cancer) (Zeng et al., 2018; Liu et al., 2020). In addition, studies also show that multiple proto-oncogenic proteins (e.g., MYC and JUN) can be degraded by the ubiquitin-proteasome system, and then function in suppressing cancer growth and progression (e.g., glioma) (Welcker and Clurman, 2008; Kim et al., 2017).

## Concluding remarks

In this review, we discuss the molecular mechanisms involved in protecting the stability and functional properties of the proteome, including molecular chaperones, ubiquitin-proteasome system, and autophagy-lysosomal system. We also describe the causes of accumulation of damaged/misfolded protein aggregates during aging, such as long-term chronic stress (e.g., oxidative stress) and dysfunctional proteolytic and repair systems, and the subsequent detrimental effects on organismal health. Current evidence suggests that improvements in cellular protein homeostasis capacity can prolong lifespan or promote healthy aging and longevity, but with a potential increase in the risk of cancer. Thus, a comprehensive understanding of the protein homeostasis network will not only shed light on the fundamental biology of aging and anti-aging, but also provide new avenues for context-dependent therapeutic interventions in various age-related diseases, including neurodegeneration and cancer.
